# Comparing the *Dictyostelium* and *Entamoeba* Genomes Reveals an Ancient Split in the Conosa Lineage

**DOI:** 10.1371/journal.pcbi.0010071

**Published:** 2005-12-16

**Authors:** Jie Song, Qikai Xu, Rolf Olsen, William F Loomis, Gad Shaulsky, Adam Kuspa, Richard Sucgang

**Affiliations:** 1 Verna and Marrs McLean Department of Biochemistry and Molecular Biology, Baylor College of Medicine, Houston, Texas, United States of America; 2 Department of Human Genetics, Baylor College of Medicine, Houston, Texas, United States of America; 3 Graduate Program in Structural and Computational Biology and Molecular Biophysics, Baylor College of Medicine, Houston, Texas, United States of America; 4 Section of Cell and Developmental Biology, Division of Biology, University of California San Diego, La Jolla, California, United States of America; University of California San Diego, United States of America

## Abstract

The Amoebozoa are a sister clade to the fungi and the animals, but are poorly sampled for completely sequenced genomes. The social amoeba *Dictyostelium discoideum* and amitochondriate pathogen *Entamoeba histolytica* are the first Amoebozoa with genomes completely sequenced. Both organisms are classified under the Conosa subphylum. To identify Amoebozoa-specific genomic elements, we compared these two genomes to each other and to other eukaryotic genomes. An expanded phylogenetic tree built from the complete predicted proteomes of 23 eukaryotes places the two amoebae in the same lineage, although the divergence is estimated to be greater than that between animals and fungi, and probably happened shortly after the Amoebozoa split from the opisthokont lineage. Most of the 1,500 orthologous gene families shared between the two amoebae are also shared with plant, animal, and fungal genomes. We found that only 42 gene families are distinct to the amoeba lineage; among these are a large number of proteins that contain repeats of the FNIP domain, and a putative transcription factor essential for proper cell type differentiation in *D. discoideum*. These Amoebozoa-specific genes may be useful in the design of novel diagnostics and therapies for amoebal pathologies.

## Synopsis

Most single-celled eukaryotes were lumped together in a single catchall classification until molecular sequencing revealed that they are a very diverse group that illustrates the different paths eukaryotic evolution has taken. Comparing a representative subset of genes indicates that one group in particular, the Amoebozoa, are a sister group to the animals and fungi, even more closely related than the plants. Despite their diversity, few simple eukaryotes have been the subject of complete genome sequencing. The genomes of two amoebozoa, *Dictyostelium discoideum* (a free-living social amoeba) and *Entamoeba histolytica* (a pathogenic amoeba), were recently completed. The authors compared the predicted proteins encoded by each organism to each other, and to other representative eukaryotes, and built a phylogenetic tree using not just a few representative genes, but the entire genomes of 23 organisms. The resulting tree closely re-created the relationships predicted from the sampled genes, including reinforcing the close relationship between the amoebozoa and the animals and fungi. The authors also found very few genes that are exclusively inherited by amoebozoa. Since some amoebozoa are important clinical pathogens, these genes are likely good targets for therapeutic agents that will not affect the animal host.

## Introduction

Comparative genomics of the bacteria and archea is well developed, has provided many insights, and has promoted the development of numerous analytical tools. The comparative genomics of eukaryotes is still in its infancy due to a relative paucity of completely sequenced eukaryotic genomes. However, genomic comparisons from species as divergent as man and the nematode *Caenorhabditis elegans* have provided important insights into the functional aspects of each genome [1]. Comparing genomes from organisms along a common evolutionary lineage and of varying phylogenetic distances has been particularly informative, and the recent sequencing and comparison of five hemiascomycete yeast genomes best illustrates this. These studies showed how the hemiascomycete lineage was shaped through the forces of massive genome duplication, reductive evolution, and gene dispersion [2]. The comparison of the first two sequenced *Drosophila* species, *D. melanogaster* and *D. pseudoobscura,* has proven so fruitful that 12 additional *Drosophila* genomes are being sequenced [3].

Although most eukaryotic genome sequencing efforts are focused on animals, fungi, and plants, the simple eukaryotes or “protists” represent a major component of the diversity of eukaryotes. Single-celled eukaryotes lack extensive fossil records, but phylogenetic trees built using exhaustive sampling of small subunit rRNA genes and selected protein coding genes have revealed a previously unappreciated diversity deep in the roots of eukaryotic ancestry [4]. Notable is the positioning of the Amoebozoa as a sister clade to the opisthokonts (animals and fungi). To date, only two Amoebozoa species have had their genomes extensively sampled, although more species are being sequenced [5]. The genome of the social amoeba *Dictyostelium discoideum* has been completely mapped and sequenced [6], and the genome of the amitochondriate human pathogen *Entamoeba histolytica* has been subjected to deep shotgun sampling and assembly into unordered scaffolds [7]. Because the Amoebozoa do not exhibit strong morphologic traits that can be used for taxonomic categorization, classification has relied heavily on sequence comparison.

Due to similarities in lifestyle, the genome of *Entamoeba* has been compared with that of other parasitic eukaryotes such as *Giardia, Trichomonas,* or *Leishmania* [7], but analyses of 100 representative genes have clustered *Dictyostelium* and *Entamoeba* as genera of a common phylum [8], each one, in turn, representing the two major arms of the Conosa lineage: the free-living Mycetozoa and the amitochondrial Archamoeba, respectively [8]. Both organisms have unusually A+T-rich genomes that have confounded sequencing and assembly, and analyses from the genomic sequences have implicated significant contributions of genes from putative horizontal gene transfer events from bacteria into the physiology of each organism [5]. We have taken advantage of having two related genomes among the Amoebozoa, and have compared the predicted proteomes of *D. discoideum* and *E. histolytica*. Although we found a sizeable number of gene families in common between the two, most of those are shared with other eukaryotes such as plants, animals, and fungi. In fact, less than 45 gene families defined the amoeba-specific proteins, which is consistent with a deep evolutionary divergence between the two amoebae as indicated by a tree constructed from the complete proteomes of 21 additional eukaryotes.

## Results

### Shared Proteins between *Dictyostelium* and *Entamoeba*

Using the complete predicted protein sets of each organism, we ran reciprocal BLASTP analyses to identify putative orthologs between *E. histolytica* and *D. discodeum,* using only proteins that hit a cognate with an e-value of ≤ 10^−5^, and requiring that each protein return its cognate from the other genome as a best hit when used as a query. This method, referred to as reciprocal best hits (RBHs), was adapted from the construction of the Clusters of Orthologous Genes (COG) database at the National Center for Biotechnology Information (NCBI) [9]. A set of 1,607 proteins passed these criteria as orthologs between *E. histolytica* and *D. discoideum;* loosening the stringency of the cutoff value did not appreciably change the number of pairs detected. To distinguish which members of this set are unique to the Amoebozoa lineage, we filtered out orthologs found also in model organisms representing plants, animals, and fungi. Using the *Homo sapiens, C. elegans, D. melanogaster, Saccharomyces cerevisiae,* and *Arabidopsis thaliana* genomes as the representative model genomes for the other sequenced eukaryotes, we determined that 1,545 of the shared orthologs between *D. discoideum* and *E. histolytica* also matched orthologs with the other major eukaryotes, with 1,199 genes being universally conserved among all seven representative eukaryotic genomes. Only 62 genes appear exclusive to the amoebozoan genomes relative to the other eukaryotes.

### Lineage-Specific Genes

The number of putative lineage-specific genes appears to be much lower among the amoebozoans than among other related species. Comparing five hemiascomycete yeast genomes identified 800 gene families out of 2,014 shared gene families [2]. However, since the species chosen were relatively closely related, the fact that a higher proportion of their genomes are shared is not surprising. Although not a perfect alternative, the divergence between *D. discoideum* and *E. histolytica* is better approximated by the greater divergence between *S. cerevisiae* and *Schizosaccharomyces pombe,* members of the Hemiascomycetes and Archaeascomycetes, respectively [10]. The Hemiascomycetes and Archaeascomycetes are major diverging branches of the Ascomycota lineage. A total of 3,281 genes in the *S. pombe* genome were described as having orthologs with the *S. cerevisiae* genome [11]. Using the *C. elegans* genome as the outgroup, 2,512 genes were predicted to be specific to the yeasts. The criteria used in this early analysis were significantly less stringent than our methodology, and having only *C. elegans* as the model outgroup significantly weakens the argument for lineage specificity. We updated the study by processing and comparing the two yeast genomes using the same RBH strategy that we used for the amoebozoan genomes. Despite the smaller proteomes of the yeast species, they share almost twice as many orthologs compared to the two amoebae (Table 1). Moreover, when compared against the five other completely sequenced model eukaryotic genomes, 372 orthologous genes were identified as being specific to these two divergent yeast species—five times more than the lineage-specific genes among the amoebae.

**Table 1:**
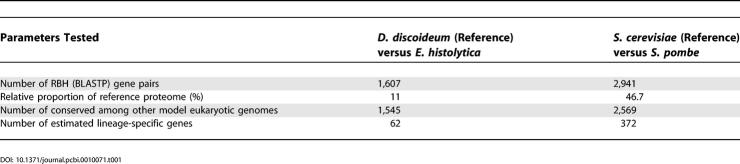
Lineage-Specific Genes in Amoebas versus Yeasts

### Shared Paralogous Families

While the RBH method is a commonly used means of identifying orthologs between two genomes, it works best when the genomes being compared are not rich in recent gene duplication events. Expanded paralog sets within each genome can confound the method, resulting in some spurious elimination of orthologous sets. While this is not an issue with relatively compact genomes such as those of prokaryotes, both the *D. discoideum* [6] and *E. histolytica* [7] genomes were shaped by significant contributions from gene duplication. We feared that missing data from the paralogs might skew the estimates for the number of lineage-specific genes. We adapted the Markov clustering algorithm [12] used in comparing the five hemiascomycete yeast lineages [2] for identifying and clustering common gene families between the two species. While Markov clustering exhibits good specificity in identifying gene families, it is best used on species of a relatively close phylogenetic distance. The sensitivity required to detect orthologs between divergent species will be overwhelmed by stronger similarity to paralogs within the same genome, and will be omitted in the clustering. We generated an optimized result by supplementing the results from the Markov clustering with groupings generated by the more sensitive RBH method. Less than 0.2% of the gene families identified by Markov clustering contradicted the RBH results. Manual inspection of some these gene families indicated that they possibly can be merged because RBH detected a structural similarity missed by the Markov clustering. Loosening the stringency of the clustering would have merged these families but would have most likely created spurious groupings as well; we considered this an acceptable error rate.

The combined results identified a set of 1,510 gene families or “archetypes” shared between the two amoeba, representing 3,216 genes in *D. discoideum* and 3,833 genes in *E. histolytica*. Of these, 1,132 gene families are shared with all the other model eukaryotes (Figure 1), with only 63 gene archetypes representing the amoeba-exclusive set. Thus, even with the inclusion of paralogs, the number of lineage-specific genes for the Conosa is remarkably small.

**Figure 1:**
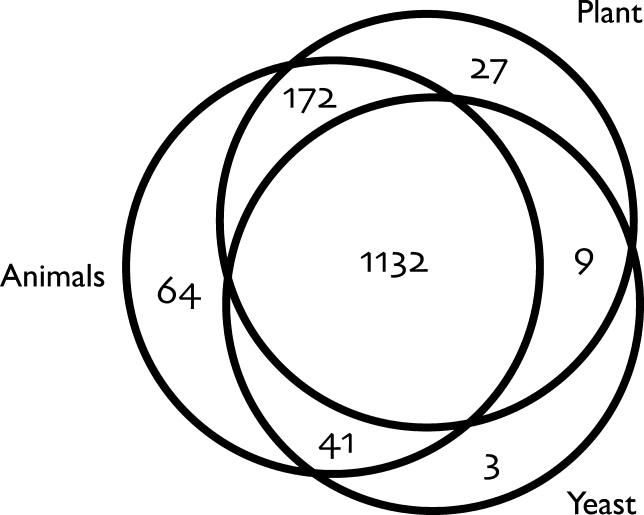
Shared Gene Archetypes between Amoeba and Other Eukaryotes The combined RBH and TribeMCL clustering identified 1,510 gene archetypes between *E. histolytica* and *D. discoideum,* with all but 63 shared with five other model eukaryotes. This Venn diagram illustrates how the shared archetypes are distributed with other eukaryotic genomes; the amoeba-specific genes are not displayed here. Animals are represented by *H. sapiens, C. elegans,* and *D. melanogaster*. Plant is represented by *A. thaliana,* and yeast by *S. cerevisiae.*

The 63 gene archetypes translate to 78 genes in the *D. discoideum* genome. We used them as queries against the NCBI nonredundant protein database (nr; as of April 2005, downloaded from http://www.ncbi.nlm.nih.gov/Database/) for matches in other organisms not represented in our model outgroups. Of these genes, 48 (representing 40 gene families) failed to match anything significant in the database. Of the remaining gene families, one matched an actin-binding protein previously identified in *Physarum polycephalum*—another Amoebozoa. A second family is enriched for proteins containing repeats of the protein domain FNIP. Until this comparison, the FNIP domain was described exclusively in *D. discoideum* and distributed among 154 proteins ranging from putative kinases to transcription factors [6]. The FNIP domain appears to be related to leucine-rich repeats, a protein motif involved in setting up protein–protein interactions [13]. In addition to the FNIP-containing proteins in *E. histolytica,* 16 FNIP-containing proteins are encoded in the genome of mimivirus, the largest virus on record [14]. Mimivirus infects *Acanthamoeba polyphaga*—itself another amoebozoan. All together, these 42 gene families are exclusively found in the Amoebozoa and represent the lineage-specific cohort of genes for this clade.

Among the remainder, we found families of ADP-ribosylglycohydrolases with an ortholog in *Neurospora crassa,* a fungus that we had not included as part of our model organism outgroup. Six of the gene families were matched primarily on the basis of alignment to a conserved domain, and not throughout the protein—we did not consider these passing criteria as orthologs, although we cannot discount the possibility that they arose from a common gene prototype. Aside from three proteins that have orthologs in *Leishmania* and *Plasmodium* (early diverging eukaryotes), the rest are orthologs retained from the prokaryotic ancestry. In no case was a match to proteins from plants or animals detected.

### Divergence between *Dictyostelium* and *Entamoeba*

Given the small number of orthologs identified that is distinctive to the lineage based on comparing five genomes, we sought to estimate the phylogenetic distance represented by *Dictyostelium* and *Entamoeba*. We had earlier used the proteome content of 17 eukaryotes to establish that *D. discoideum* had diverged later from the opisthokont lineage than the plants did [6]. Supplementing the data with the proteomes of four organisms in addition to *E. histolytica,* the expanded tree demonstrates that the divergence between *E. histolytica* and *D. discoideum* is even deeper than between the animals and fungi (Figure 2). Note that, although the revised tree used significantly more data in its construction, the topology is essentially identical to the tree built using 100 sample genes [8], and remains unchanged with regards to the divergence of the Amoebozoa from the opisthokonts as a later event than the divergence of plants from that lineage.

**Figure 2:**
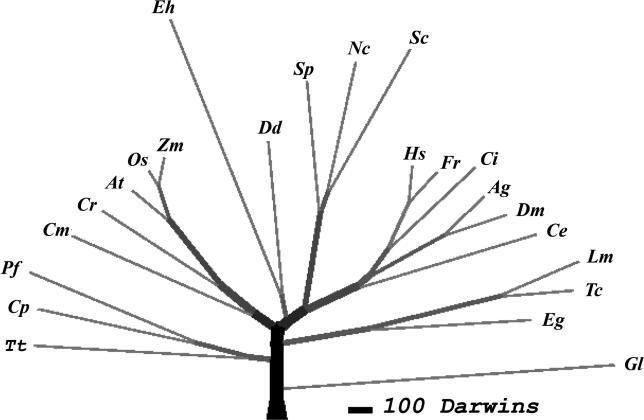
Proteome-Based Phylogeny of Eukaryotes Abbreviations for organisms are as follows: Ag, *A. gambiae;* At, *A. thaliana;* Ce, *C. elegans;* Cr, *C. rheinhardtii;* Ci, *C. intestinales;* Cp, *C. parvum;* Cm, *C. morolae;* Dd, *D. discoideum;* Dm, *D. melanogaster;* Eg, *E. gracilis;* Eh, *E. histolytica;* Fr, *F. rubripes;* Gl, *G. lamblia;* Hs, *H. sapiens;* Lm, *L. major;* Nc, *N. crassa;* Os, *O. sativa;* Pf, *P. falciparum;* Sc, *S. cerevisiae;* Sp, *S. pombe;* Tt, *T. thermophila;* Tc, *T. cruzi;* and Zm, *Z. mays.* 1 Darwin = 1/2000 of the divergence between *S. cerevisiae* and *H. sapiens*. Branch thickness is proportional to the size of each clade. The tree was constructed by full maximum likelihood with clusters of orthologs generated from whole proteomes from each of the organisms. A phylogeny program used for constructing a new amino acid replacement model (23) determined the individual nodes and branch lengths.

### Universal Common Eukaryotic Genes

Given the phylogenetic distances represented by the seven model organism genomes in this comparison, the 1,132 gene families that are shared among all the model eukaryotes may define a core set of eukaryotic genes. Using *D. discoideum* as the reference genome for this analysis, we chose to explore the available annotations in this organism. The 2,726 *D. discoideum* genes represented by the “universal” ortholog set were enriched for Gene Ontology (GO) terms [15] as a consequence of receiving functional assignments extrapolated from the study of the orthologs in other organisms. The relative enrichment of GO terms in these annotations permitted the use of the automated GOAT tool [16] to recognize the major functions of this collection of proteins. The full results are available from the Supporting Information. As expected, the genes in this group encode proteins that regulate and propagate the cell cycle, components of DNA replication, RNA transcription, and protein synthesis, as well as a significant number predicted to be involved in cellular transport. Nevertheless, 257 of the orthologous eukaryotic gene families have no GO annotation.

### Horizontal Gene Transfer

Horizontal gene transfer (HGT) is a major force in the evolution of prokaryotic genomes [17], but its impact on eukaryotic genomes is not as easily detected or determined. Basic methods for identifying HGT candidates rely on seeking out proteins or protein domains in a eukaryotic genome that are statistically predicted to have a bacterial source (and can be eliminated from possible contamination during the course of genome sequencing) and are unlikely to have been inherited from the ancestor. Significant numbers of HGT candidates were reported in both *D. discoideum* and *E. histolytica* annotations, all of them reportedly contributing strongly to the physiology of each organism. However, the methods used to identify HGT candidates were peculiar to the respective organisms.

Comparing the gene content of two related species can serve to detect false positives among the putative products of HGT—a gene that was acquired through recent lateral transfer is unlikely to share an ortholog in a relatively closely related organism. Of the 18 HGT candidates identified from the annotation of the *D. discoideum* genome, only one, DDB0204031, annotated as a beta-eliminating lyase, was found among the 1,510 orthologous gene families in common with *E. histolytica.* This gene is likely to have been inherited xenologously.

## Discussion

Given the relative paucity of sequenced genomes among the Amoebozoa, the availability of two sampled genomes presents an important first look at the distinctive physiology and evolution of this sister clade to the opisthokonts. The two representative organisms in this clade, *E. histolytica* and *D. discoideum,* despite dramatic differences in physiology and life modes, share distinct similarities on the genome scale. Both genomes are extremely A+T rich, which led to great difficulties in sequencing and assembly, and both genomes are also relatively gene rich, with small predicted introns. We have identified 1,132 gene families conserved across seven genomes, representing the major phyletic branches of the eukaryotes: *D. discoideum, E. histolytica, H. sapiens, D. melanogaster, C. elegans, A. thaliana,* and *S. cerevisiae*. The gene families in this collection fall into expected categories: proteins known to be involved in housekeeping functions such as transcription, translation, and replication. However, a significant number of genes involved in organogenesis, cell migration, and environmental response are conserved across all these diverse phyla, even in organisms that do not form organs, or are nonmotile.

The 1,132 “universally” conserved orthologs represent 1,967 genes in the *S. cerevisiae* genome; we cross-referenced this list against the list of 1,189 genes essential for growth on rich medium [18]. While the number of essential genes in yeast comprises 18.8% of the 6,298 genes in the *S. cerevisiae* genome, 667 of them are among the conserved orthologs. This represents enrichment to 34%, indicating that the genes in this set are ancient, conserved gene archetypes that may serve fundamental functions in all eukaryotes. However, 523 of the yeast orthologs to “universally” conserved genes are not vital. Moreover, 257 of the conserved gene families as yet do not have GO assignments. Elucidation of their functions will have profound implications for our understanding of all eukaryotes. When the predicted proteomes from each amoeba genome are compared, we find 1,510 orthologous gene families, but only 63 of these families were not found among the five model eukaryotic genomes we had chosen. More detailed inspection revealed that 42 of these families appear to be exclusively carried by amoebae, and most of the rest are ancient genes retained from prokaryotes. The very small number of Amoebozoa lineage-specific genes was surprising; we entertained the possibility that it could be an artifact of differences in gene prediction algorithms. The methods used in this comparison relied on using the predicted protein sequences of each genome project, and trusting that each respective project has chosen the appropriate criteria peculiar to that organism to generate the best possible predictions. Orthologs will not be found if they are not predicted as coding regions in one or the other organism. The exon-dense nature of both genomes makes this scenario unlikely. However, expansion of the comparison into undetected open reading frames of each respective genome can prove useful in detecting hidden lineage-specific orthologs. A preliminary scan of the noncoding regions of the *D. discoideum* region using TBLASTN has yielded nothing more than a few pseudogenes (unpublished data), so we do not think that the differences in gene prediction algorithms had a major effect on this estimate. Alternatively, the physiology of the Amoebozoa may be more strongly influenced by RNA-based effectors than other eukaryotes. Earlier scans for short noncoding RNAs in *D. discoideum* identified novel species unreported in other organisms [19]. Perhaps a closer inspection of the nonprotein coding regions of the genome will unearth conserved motifs indicative of strongly conserved RNA-based physiology distinct from other eukaryotes. Barring these alternative explanations, anything distinctive in the physiology of the Conosa lineage, if not for the entire Amoebozoa, lies among these 42 lineage-specific gene families.

The construction of an expanded phylogenetic tree using the complete proteomic content of 23 eukaryotic genomes yielded a general topology that is essentially identical to the earlier grouping of *D. discoideum* and *E. histolytica* as sharing a common ancestor [8], but the distances indicate a divergence almost as ancient as that between fungi and animals. Since these two represent but one subphylum of the Amoebozoa, this suggests that the diversity among this clade is very large indeed. Sequencing additional genomes from this clade will undoubtedly return rich veins of information about presently unexplored physiology.

In *D. discoideum,* 51 genes represent the 42 amoeba-specific gene families. Three other genes are found among only the amoebae and two pathogenic primitive eukaryotes, *Leishmania* and *Plasmodium*. For most of these genes, we cannot draw from studies in orthologs found in other organisms to interpret their functional roles due to their lineage-specific nature. We can, however, infer the putative functions of these genes based on structural features, and independent mutagenesis experiments. Only three of the lineage-specific genes have been mutated among the more than 900 genes that are being systematically mutated in *D. discoideum* (unpublished data and personal communication, http://www.dictybase.org), and one of these is the putative transcription factor *cudA,* which is necessary for the entry of *D. discoideum* into terminal differentiation [20]. Given that *E. histolytica* lacks a multicellular stage but retains an ortholog argues that *cudA* has a more vital role in most amoebae beyond regulating social behavior and cell-type differentiation. Other amoeba-specific genes include a histidine kinase gene family, a bZIP transcription factor, and a calcium-binding protein of unknown function. These lineage-specific genes may represent the distinctive physiologic elements of all amoebae. Future experiments into these key genes using the easily tractable and nonpathogenic *D. discoideum* can be extrapolated to the physiology of the *E. histolytica,* an important human pathogen with more laborious culturing requirements [21]. These proteins are likely to be the best substrates for drugs that target *E. histolytica,* as well as other pathogenic amoebae such as *Acanthamoeba,* without affecting the vertebrate host.

## Materials and Methods

### Comparison algorithms.

All work was done based on version 2.0 of the *D. discoideum* genome (http://www.dictybase.org) and the latest release of the *E. histolytica* genome as of April 2005. RBH was performed by generating BLAST databases from the predicted proteins in each respective genome, and performing a BLASTP analysis (NCBI BLAST v2.2.1), using the following parameters: sequence filtering by SEG with default settings; Matrix BLOSUM62; gap opening penalty = 11; and gap extension penalty = 1. The minimum high-scoring segment pair (HSP) was set at 50 residues, and the minimum identity of the longest HSP was set at 20%. Results were filtered for hits with e-value scores less than 10^−5^. A successful RBH ortholog returns the same protein as the best hit when the query is reversed, and the querying genome is now used as the subject database. The same parameters were used in updating the comparison between *S. cerevisiae* and *S. pombe*. Sources for the relevant genome databases are listed and linked in the Web site provided in the Supporting Information.

Clustering into gene families used a modification of the TribeMCL-based method described in Dujon et al. (http://www.ebi.ac.uk/research/cgg/tribe/) [2]. Briefly, both protein databases were pooled and subjected to an all-versus-all BLASTP comparison, with the minimum identity of the longest HSP set at 25%, and the required HSP length at least 50% of the query length (all other parameters were as described above). We had empirically determined these cutoff parameters that would maximize overlap with the RBH results and minimize inclusions due to alignments of short HSPs. The results were processed into gene families using TribeMCL, with the parameter “Inflation = 4.0.” This is the default value; increasing or decreasing it did not affect the composition or number of proteins being clustered appreciably, only how they were grouped. The final set of orthologous gene families is the union of the TribeMCL and RBH results; where TribeMCL clustering “broke” an RBH pairing, we retained the TribeMCL families. The set of *D. discoideum* genes represented by gene families excluded from overlaps with the model animal, plant, and yeast outgroups (putatively “amoeba-specific”) were in turn compared via BLASTP against the NCBI nonredundant database to compare against all other organisms not used in the set of model eukaryotic genomes, and manually inspected to categorize them into appropriate bins. Custom Perl and Unix shell scripts were written to parse results as necessary.

### Construction of phylogenetic tree.

The phylogenetic tree is an expansion of the construction described in Eichinger et al. [6]. Tree rooting was done with a set of clustered orthologs generated from the proteomes of the seven archaea *(Aeropyrum pernix, Archaeoglobus fulgidus, Halobacterium sp., Pyrococcus abyssi, Methanococcus jannaschii, Sulfolobulus solfataricus,* and *Thermoplasma acidophilum)* by the COG methodology [9]. The phylogenetic relationships of these Archaea were previously established [8]. The clusters were BLAST aligned [22] against the proteins of eight eukaryotes: *A. thaliana* (At), *Oryza sativa* (Os), *S. cerevisiae* (Sc), *S. pombe* (Sp), *D. melanogaster* (Dm), *Anopheles gambiae* (Ag), *H. sapiens* (Hs), and *Fugu rubripes* (Fr). Proteins that could be easily aligned over more than half their length were considered appropriate for rooting. Rooting was done with a set of 159 clusters with at least one member from each of the major groups: plants, fungi, and animals. All possible root positions among these groups were tested. Bootstrap values were highest for rooting in the interval between plants and fungi, with animals diverging after yeast (96/100). The second-highest values were found for the interval between yeast and animals, with plants diverging after animals. The positions of a chordate, *Ciona intestinales* (Ci), another fungus, *N. crassa* (Nc), a nematode, *C. elegans* (Ce), and corn, *Zea mays* (Zm), were then established by maximum likelihood on databases of clusters of likely orthologs or evolutionary clusters of orthologs (ECOs) generated by a multimatrix model of protein divergence [23]. The favored position of the archaebacterial root for this set of 12 organisms remained at the junction of plants and fungi (93/100). We then tested the position of the malarial parasite, *Plasmodium falciparum* (Pf), and found that it diverged before the plant/fungal split. When the archaebacterial root was determined with this set of 13 organisms, the interval between plants and fungi received 100/100 bootstraps with *Plasmodium* as an early diverging organism. The root position was then fixed. *D. discoideum* (Dd), *Leishmania major* (Lm), *Giardia lamblia* (Gl), and *Chlamydomonas rheinhardtii* (Cr) were then added to the tree [6]. Using the same approaches, we added the red alga *Cyanidioschyzon morolae* (Cm), the alveolates *Cryptosporidium parvum* (Cp), *Tetrahymena thermophila* (Tt), and the euglenoids *Trypanosoma cruzi* (Tc) and *Euglena gracilis* (Eg). The positions of the nodes for the newly added organisms were all supported by 100/100 bootstraps. Finally, the position of *E. histolytica* was determined using the complete set of 5,908 ECOs. The length of the *Entamoeba* branch was computed with 987 ECOs.

## Supporting Information

We have made all gene lists and raw BLAST reports available for query and download at http://dictygenome.org/supplement/rsucgang/song_2005. Analyses about the HGT candidates between *D. discoideum* and *E. histolytica,* and the clustering of the “universally” conserved orthologs in *D. discoideum,* are also available for download.

### Accession Numbers

The InterPro (http://www.ebi.ac.uk/interpro) accession number for the protein motif FNIP is IPR008615, and for LRR1 is IPR001611.

## Abbreviations

GO: Gene Ontology
HGT: horizontal gene transfer
HSP: high-scoring segment pair
NCBI: National Center for Biotechnology Information
RBH: reciprocal best hit
